# Patient‐Reported Dysphagia After Multimodal Therapy and Radial Forearm Free‐Flap Reconstruction for OPSCC: Associations With FEES Findings, Psychological Distress, and Xerostomia

**DOI:** 10.1002/lio2.70617

**Published:** 2026-10-20

**Authors:** Inga Marte Charlott Seuthe, Julius Friedrich Ennenbach, Natalja Bolotina, Nermin Rüdiger, Sabine Eichhorn, Eren Erdogan, Stijn Bogaert, Stefan Dazert, Steffen Maune, Jonas Jae‐Hyun Park

**Affiliations:** ^1^ Department of Otorhinolaryngology, Head and Neck Surgery, Catholic Hospital Hagen University of Witten/Herdecke Hagen Germany; ^2^ Department of Otorhinolaryngology, Head and Neck Surgery Hospitals of the City of Cologne Cologne Germany; ^3^ Department of Otorhinolaryngology, Head and Neck Surgery, St. Elisabeth‐Hospital Ruhr University Bochum Bochum Germany

**Keywords:** dysphagia assessment, fiberoptic endoscopic evaluation of swallowing, oropharyngeal squamous cell carcinoma, patient‐reported outcome measures, swallowing‐related quality of life

## Abstract

**Objective:**

This study investigated whether fiberoptic endoscopic evaluation of swallowing (FEES)‐derived parameters reflect patient‐reported dysphagia and swallowing‐related quality of life in patients with oropharyngeal squamous cell carcinoma (OPSCC) after surgery‐based multimodal therapy with free‐flap reconstruction. In addition, it assessed the roles of psychological distress and xerostomia in this context.

**Methods:**

This multicenter, cross‐sectional exploratory study included 50 patients. FEES parameters comprised Penetration–Aspiration Scale (PAS) and residue scores. Patient‐reported outcomes were assessed using the Sydney Swallow Questionnaire (SSQ) and MD Anderson Dysphagia Inventory (MDADI), psychological distress using the Hospital Anxiety and Depression Scale (HADS), and xerostomia using the Xerostomia Inventory (XI). Associations were examined using Spearman correlations with Benjamini–Hochberg correction and exploratory rank‐transformed multiple linear regression.

**Results:**

FEES parameters showed no significant bivariate associations with SSQ or MDADI total scores after correction. HADS and XI showed moderate to strong bivariate associations with SSQ and MDADI. In exploratory multivariable analyses, HADS (*β* = 0.372, *p* = 0.012) and PAS max (*β* = 0.318, *p* = 0.006) were independently associated with SSQ, whereas XI was not (*β* = 0.268, *p* = 0.065). HADS (*β* = −0.339, *p* = 0.008), XI (*β* = −0.433, *p* < 0.001), and PAS max (*β* = −0.301, *p* = 0.003) were independently associated with MDADI.

**Conclusion:**

The FEES‐derived parameters only partially reflected patient‐reported swallowing impairment. These findings support a multidimensional understanding of post‐therapeutic dysphagia and suggest routine integration of psychosocial and symptom‐oriented assessments into follow‐up care in this specific patient population.

**Level of Evidence:**

4.

**Trial Registration:**

German Clinical Trials Register (DRKS) Registration Number: DRKS00038870

## 1 Introduction

The incidence of oropharyngeal squamous cell carcinoma (OPSCC) has increased worldwide in recent years [1]. The most important risk factors for the development of OPSCC include tobacco use, excessive alcohol consumption, and infection with human papillomavirus (HPV) [2, 3]. Despite advances in multimodal therapy, OPSCC is associated with high functional morbidity [4]. In particular, dysphagia represents one of the most common and burdensome long‐term sequelae following multimodal therapy with flap reconstruction. Surgical treatment involving tumor resection and reconstructive surgery is associated with complex functional changes in swallowing. In addition, adjuvant therapy is usually required thereafter, which additionally affects swallowing function. Persistent swallowing disorders can have a lasting negative impact on nutrition, social participation, and health‐related quality of life [5, 6].

Fiberoptic endoscopic evaluation of swallowing (FEES) or videofluoroscopic swallow study (VFSS) are considered the established standard for the objective assessment of oropharyngeal swallowing in patients with head and neck squamous cell cancer (HNSCC) [7, 8]. Previous studies have demonstrated that associations between instrumental swallowing findings and subjectively perceived dysphagia are only partial and inconsistent [9, 10, 11, 12, 13]. However, these studies largely included heterogeneous HNSCC populations comprising different primary tumor sites and treatment modalities. Patients with OPSCC undergoing tumor resection followed by microvascular free‐flap reconstruction represent a more specifically defined functional subgroup, in whom swallowing impairment may reflect the combined effects of oropharyngeal resection, reconstruction‐related changes in anatomy and function, and subsequent adjuvant treatment. Therefore, findings derived from broader HNSCC populations may not fully reflect the relationship between instrumental swallowing findings and patient‐reported swallowing impairment in this specific treatment setting.

Accordingly, the present exploratory study investigated the associations between FEES‐based swallowing findings and patient‐reported dysphagia in patients with OPSCC after surgery‐based multimodal therapy with microvascular free‐flap reconstruction. We hypothesized that FEES‐derived measures of swallowing impairment would be associated with patient‐reported dysphagia. Secondary analyses examined associations with swallowing‐related quality of life and explored the potential contributions of psychological distress and xerostomia to patient‐reported swallowing outcomes.

## 2 Materials and Methods

### 2.1 Study Population

In this multicenter, cross‐sectional exploratory study with retrospective and prospective components, patients with histologically confirmed OPSCC were included if they had undergone primary surgical treatment and free‐flap reconstruction followed by adjuvant therapy. All participants completed oncological treatment at least 3 months before study data collection. All assessments were performed during routine follow‐up visits. For the retrospective component, study‐relevant assessments, including FEES and patient‐reported outcome measures (PROMs), had been performed before formal study initiation as part of routine oncological follow‐up according to an established institutional protocol. At the time of these routine assessments, patients had provided consent for the use of their clinical data for research purposes. These data were included in the present study only after approval by the ethics committee had been obtained. For the prospective component, patients were recruited after ethics approval and underwent the same assessments during routine oncological follow‐up. No additional study‐specific diagnostic procedures or deviations from the established follow‐up protocol were performed.

Recruitment was conducted at two German Cancer Society (DKG)‐certified ENT centers (Department of Otorhinolaryngology, University Witten/Herdecke at the Catholic Hospital Hagen; Department of Otorhinolaryngology, Hospitals of the City of Cologne) between December 2025 and July 2026. The study was approved by the ethics committee of University Witten/Herdecke (ethics approval number: S‐322/2025) and was conducted in accordance with the latest version of the Declaration of Helsinki. Furthermore, the study was registered in a clinical trial registry (registration number: DRKS00038870).

### 2.2 Inclusion and Exclusion Criteria

Study participation required, in addition to the criteria mentioned above, that all included patients underwent a standardized FEES according to the study protocol. Moreover, all questionnaires had to be completed in full. The interval between FEES and completion of the questionnaires was not allowed to exceed 14 days. Patients also had to be capable of reliably cooperating during FEES and of independently completing the questionnaires used in the study. Patients were excluded if they had tumor recurrence or a second primary malignancy in the head and neck region at the time of data collection, or if they were treated with palliative intent. Patients with pre‐existing or concomitant neurological disorders known to affect swallowing function, including neurodegenerative or neuromuscular diseases, were also excluded, as were patients with non‐oncologic dysphagia prior to tumor treatment. The absence of pre‐existing non‐oncologic dysphagia was determined based on the available medical history and clinical records. Additional exclusion criteria included cognitive impairment or language barriers that precluded reliable performance of FEES or independent completion of the questionnaires.

### 2.3 PROMs

Validated PROMs were used to assess patient perception of swallowing function, swallowing‐related quality of life, psychological distress, and xerostomia. All questionnaires were completed independently by the patients.

#### 2.3.1 Sydney Swallow Questionnaire (SSQ)

Subjective swallowing function was assessed using the SSQ (German version). This is a validated, dysphagia‐specific questionnaire. It is used to assess symptom‐related oropharyngeal swallowing difficulties. Higher scores indicate a more severe subjective swallowing impairment [14].

#### 2.3.2 M. D. Anderson Dysphagia Inventory (MDADI)

Swallowing‐related quality of life was assessed using the German version of the MDADI. This questionnaire is used to evaluate the impact of dysphagia on swallowing‐related quality of life in patients with head and neck cancer and includes global, emotional, functional, and physical domains. Higher scores indicate better swallowing‐related quality of life [15].

#### 2.3.3 Hospital Anxiety and Depression Scale (HADS)

Psychological distress was assessed using HADS. This is a validated instrument for measuring symptoms of anxiety and depression in patients with somatic illnesses and comprises two 7‐item subscales: HADS‐A for anxiety and HADS‐D for depression [16]. Higher scores indicate greater psychological symptom burden. HADS‐A and HADS‐D were analyzed separately, and the HADS total score was used as an overall measure of psychological distress.

#### 2.3.4 Xerostomia Inventory (XI)

Xerostomia was assessed using XI. The XI is used for the quantitative evaluation of xerostomia‐related symptoms. Higher scores indicate a greater severity of xerostomia [17].

### 2.4 FEES

Objective swallowing function was assessed using FEES according to the standardized institutional FEES protocol used in clinical practice. Where clinically feasible, the examination comprised at least five individual swallows of thin liquid, three spoonfuls of pureed consistency of approximately 5 mL each, and at least two bites of solid consistency. This structured assessment across repeated trials and different bolus consistencies is consistent with published FEES approaches used in patients with head and neck cancer, including the DIGEST‐FEES framework [18].

FEES recordings were independently evaluated by two raters who were blinded to all patient‐reported outcome scores. In cases of disagreement, the recordings were reviewed jointly, and a final score was determined by consensus.

Penetration and aspiration were evaluated using the Penetration–Aspiration Scale (PAS) [19]. Analyses were performed in a consistency‐specific manner, and the highest PAS score observed was used for further analysis. Pharyngeal residue was assessed using the Yale Pharyngeal Residue Severity Rating Scale (YPRSRS) for the vallecula and piriform sinuses [20]. In addition, residue burden and clearance were evaluated using the Boston Residue and Clearance Scale (BRACS) [21]. In some patients (*n* = 2), not all bolus consistencies could be tested due to aspiration risk or safety concerns. These cases were retained in the analysis, and the highest scores observed for each parameter during the completed portions of the examination were used. This approach was chosen to avoid excluding patients with more severe swallowing impairment.

### 2.5 Statistics

No formal a priori power or sample‐size calculation was performed. A target sample size of 50 patients was prespecified based on recruitment feasibility in this highly selected OPSCC population and the exploratory nature of the study, with the aim of obtaining preliminary estimates of moderate correlations and corresponding confidence intervals. The recruitment target was achieved.

Statistical analyses were performed using SPSS 29.0 (IBM Corp., Armonk, NY, USA). Descriptive statistics are presented as median and interquartile range or mean ± standard deviation, as appropriate.

Associations between objective swallowing parameters, patient‐reported outcomes, psychological distress, and xerostomia were assessed using Spearman rank correlation coefficients. Correlation strength was classified as very weak (|*ρ*| < 0.20), weak (0.20 ≤ |*ρ*| < 0.40), moderate (0.40 ≤ |*ρ*| < 0.60), strong (0.60 ≤ |*ρ*| < 0.80), and very strong (|*ρ*| ≥ 0.80) [22]. To account for multiple testing, *p* values were adjusted using the Benjamini–Hochberg false discovery rate procedure [23]. The primary family comprised eight correlations between the four FEES‐based parameters and SSQ and MDADI. The secondary family comprised the eight correlations of HADS‐A, HADS‐D, HADS total, and XI with SSQ and MDADI. The tertiary family comprised the 16 correlations of HADS‐A, HADS‐D, HADS total, and XI with the four FEES‐based parameters. Statistical significance for the correlation analyses was determined based on FDR‐adjusted *p* values, with *p*_FDR_ < 0.05 considered statistically significant.

Additional exploratory analyses were performed to assess potential sources of variation in patient‐reported swallowing outcomes. Spearman rank correlations were calculated between receipt of speech and language therapy (SLT; yes/no) and SSQ and MDADI total scores, as well as between time since completion of oncological treatment and SSQ total score. These analyses were not included in the predefined FDR correction families, and no additional adjustment for multiple testing was applied.

To identify independent associations of subjective dysphagia (SSQ) and swallowing‐related quality of life (MDADI), rank‐transformed multiple linear regression analyses were performed to account for the non‐normal distribution of outcome variables. The Penetration–Aspiration Scale (PAS max) was selected a priori as the primary FEES‐based parameter for regression analyses, as it represents a well‐established and clinically relevant measure of airway invasion and swallowing safety. To avoid overfitting in this exploratory cohort, additional FEES‐based parameters were not simultaneously included in the regression models. Regression coefficients are presented with 95% confidence intervals and nominal *p* values. No additional multiplicity adjustment was applied to the regression coefficients, and these results should therefore be interpreted as exploratory.

Figures were prepared using GraphPad Prism version 8.0 (GraphPad Software, San Diego, CA, USA) and Microsoft Word.

## 3 Results

### 3.1 Patient Cohort

A total of 50 patients were included in the study between December 2025 and July 2026. The interval between completion of oncological treatment and study data collection ranged from 3 to 112 months. An overview of the screened and included patients is shown in Figure 1. A detailed breakdown of the patient cohort is provided in Table 1.

**FIGURE 1:**
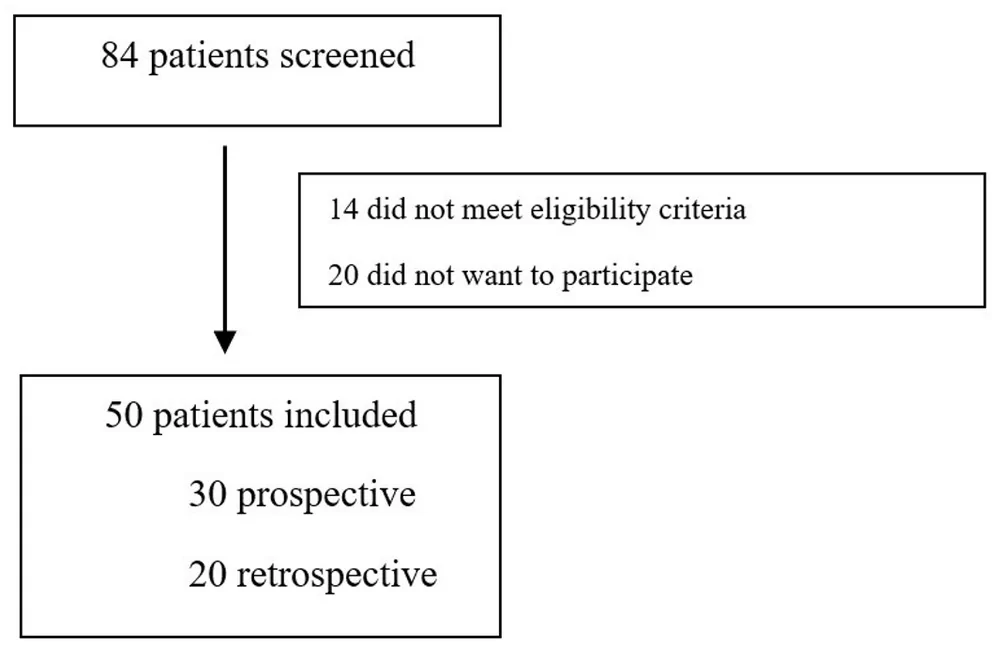
Flow diagram of patient screening, exclusion, and final study population.

**TABLE 1:** Clinicopathological characteristics of the OPSCC patients.

Characteristic	*N*	%	*N* (total)
Sex			50
Male	37	74.0	
Female	13	26.0	
Age			
Median	63		
Range	47–82		
T‐stage			50
1	13	26.0	
2	26	52.0	
3	9	18.0	
4	2	4.0	
N‐stage			50
0	6	12.0	
1	25	50.0	
2	16	32.0	
3	3	6.0	
M‐stage			50
0	50	100.0	
1	0	0.0	
AJCC eighth edition			50
I	25	50.0	
II	12	24.0	
III	4	8.0	
IV	9	18.0	
Localization of flap^a^			68
Tonsillar region	34	50.0	
Base of tongue	24	35.3	
Soft palate	10	14.7	
Type of flap reconstruction			50
Radial forearm free flap (RFFF)	50	100.0	
Treatment			50
Surgery + RT	14	28.0	
Surgery + RCT	36	72.0	
Surgery + RT + CTX	0	0.0	
Feeding status at time of FEES			50
No oral intake	0	0.0	
PEG feeding with occasional trial sips/bites	2	4.0	
PEG feeding with regular oral intake	4	8.0	
Fully oral intake of a single consistency	0	0.0	
Fully oral intake of multiple consistencies with assistance	9	18.0	
Fully oral intake of multiple consistencies without assistance, with avoidance of certain foods	13	26.0	
Fully oral intake without restrictions	22	44.0	
Tracheostomy at time of FEES			50
+	6	12.0	
−	44	88.0	
SLT for dysphagia			50
+	26	52.0	
−	24	48.0	
Duration of SLT prior to FEES (months)			
Median	1.5		
Range	0–36		
p16‐status			50
+	38	76.0	
−	12	24.0	

Abbreviations: FEES = fiberoptic endoscopic evaluation of swallowing, PEG = percutaneous endoscopic gastrostomy, RCT = radiochemotherapy, RT = radiotherapy, RT + CTX = radiotherapy with cetuximab, SLT = speech and language therapy.

^a^ Multiple selections allowed.

### 3.2 PROMs and FEES Findings

PROMs and objective swallowing parameters as assessed by FEES are summarized in Table 2 and Table 3. PROMs demonstrated considerable variability. The median SSQ total score was 501.0 (IQR: 206.25–785.25), indicating a wide range of subjective swallowing impairment. Swallowing‐related quality of life, assessed by MDADI, was moderately reduced overall, with a median total score of 75.50 (IQR: 57.75–90.00). Subscale analysis showed higher functional scores compared to physical and emotional domains. Psychological distress, measured by HADS, was low to moderate, with a median total score of 7.50 (IQR: 2.75–12.00). Xerostomia scores were in the moderate range, with a median XI score of 33.00 (IQR: 26.00–39.25).

**TABLE 2:** Objective swallowing function assessed by FEES.

FEES parameter	Scale (range)	Median (IQR) 25–75	Min–max
Airway invasion			
PAS (max)	1–8	2 (1–4)	1–8
Pharyngeal residue			
YPRSRS—vallecula (max)	0–4	4 (3–4)	1–4
YPRSRS—piriform sinus (max)	0–4	3 (1–3.25)	0–4
Residue burden and clearance			
BRACS (max)	0–10	7 (5–8)	0–10

*Note:* Higher scores indicate worse swallowing function.

Abbreviations: BRACS = Boston Residue and Clearance Scale, PAS = Penetration–Aspiration Scale, YPRSRS = Yale Pharyngeal Residue Severity Rating Scale.

**TABLE 3:** PROMs of the OPSCC patients.

PROM	Score range	Median (IQR)	Min–max
Subjective swallowing function			
SSQ—total score	0–1700	501.0 (206.25–785.25)	74–1558
Swallowing‐related quality of life			
MDADI—global score	20–100	70.00 (40.00–80.00)	20–100
MDADI—emotional subscale	20–100	73.33 (56.67–86.67)	43–100
MDADI—functional subscale	20–100	80.00 (59.00–93.00)	24–100
MDADI—physical subscale	20–100	67.50 (57.50–88.13)	25–100
MDADI—total score	20–100	75.50 (57.75–90.00)	33–99
Psychological distress			
HADS—anxiety (HADS‐A)	0–21	5.00 (1.00–7.75)	0–16
HADS—depression (HADS‐D)	0–21	3.00 (1.00–6.00)	0–12
HADS—total score	0–42	7.50 (2.75–12.00)	0–27
Xerostomia			
XI	11–55	33.00 (26.00–39.25)	13–55

*Note:* Higher SSQ, HADS, and XI scores indicate greater symptom burden, whereas higher MDADI scores indicate better swallowing‐related quality of life.

Abbreviations: HADS, Hospital Anxiety and Depression Scale; MDADI, MD Anderson Dysphagia Inventory; SSQ, Sydney Swallow Questionnaire; XI, Xerostomia Inventory.

### 3.3 Association Between FEES and Subjective Swallowing Outcomes

PAS max showed weak nominal correlations with higher SSQ total scores (*ρ* = 0.344, unadjusted *p* = 0.014) and lower MDADI total scores (*ρ* = −0.323, unadjusted *p* = 0.022). However, neither association remained statistically significant after Benjamini–Hochberg adjustment (SSQ: *p*_FDR_ = 0.088, MDADI: *p*_FDR_ = 0.088). No significant associations were observed for the residue‐based FEES parameters. The overall association pattern is shown in Figure 2.

**FIGURE 2:**
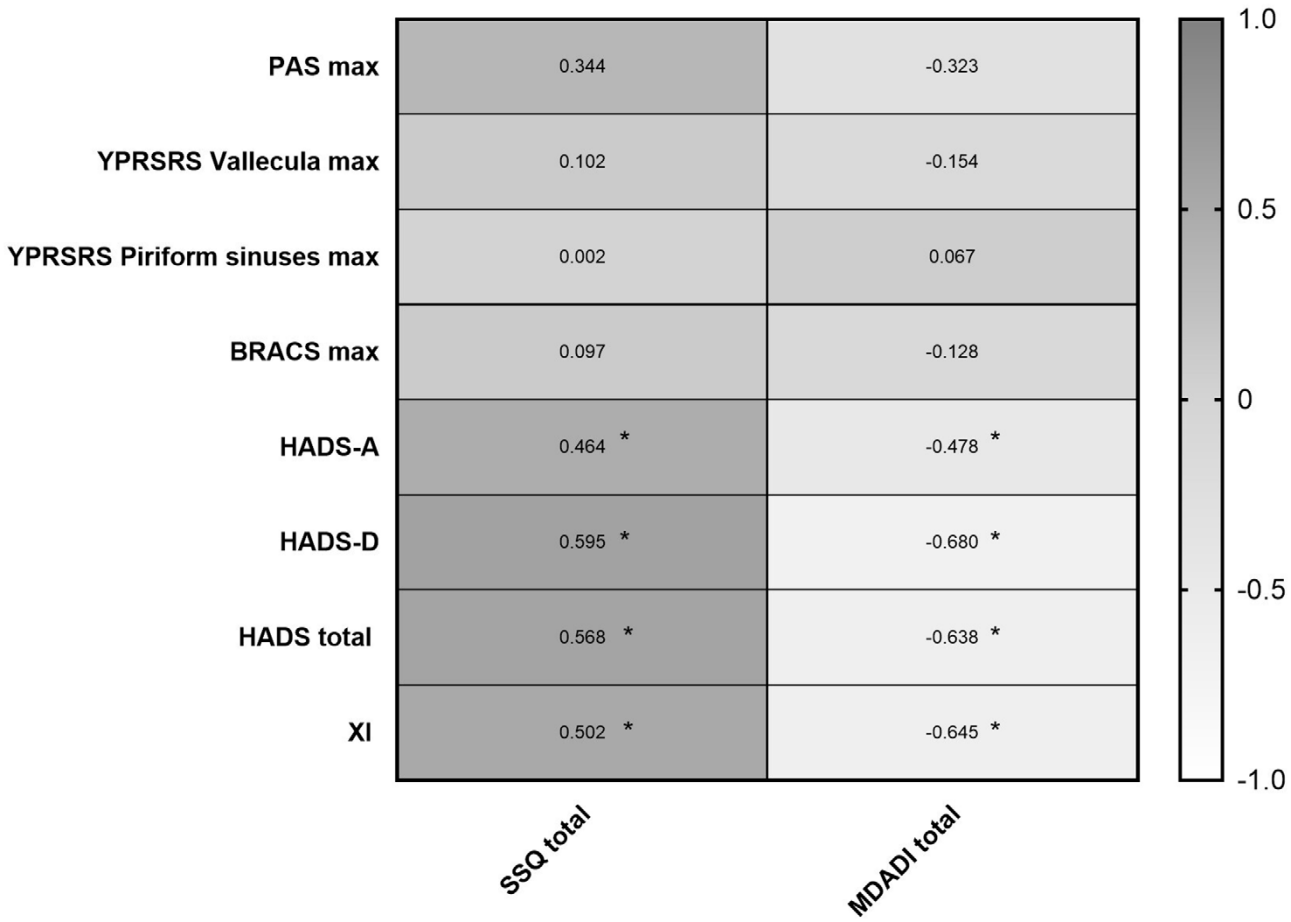
Association heatmap of FEES‐based swallowing parameters, psychological distress, xerostomia, and patient‐reported swallowing outcomes. Spearman correlation heatmap showing associations between FEES‐based parameters, HADS scores, xerostomia, and patient‐reported swallowing outcomes. Values indicate Spearman's *ρ*. Higher SSQ scores indicate greater subjective dysphagia, whereas higher MDADI scores indicate better swallowing‐related quality of life. *p_FDR_ < 0.001.

### 3.4 Association of HADS and Xerostomia With PROMs

Psychological distress and xerostomia showed moderate to strong associations with patient‐reported outcomes. HADS scores demonstrated significant positive associations with SSQ total scores (ρ = 0.464–0.595, all *p*_FDR_ < 0.001), suggesting increased subjective dysphagia with higher levels of psychological distress. Correspondingly, HADS scores were significantly negatively associated with MDADI total scores (*ρ* = −0.478 to −0.680, all *p*_FDR_ < 0.001), consistent with reduced swallowing‐related quality of life with increasing psychological burden. XI also showed significant association with both subjective measures, with a positive association with SSQ (*ρ* = 0.502, *p*_FDR_ < 0.001) and a negative association with MDADI (*ρ* = −0.645, *p*_FDR_ < 0.001). The overall association pattern is summarized in Figure 2.

### 3.5 Association of HADS and Xerostomia With Objective Swallowing Parameters (FEES)

No consistent associations were observed between psychological distress, xerostomia, and objective FEES‐based swallowing parameters. Correlation coefficients were generally very weak to weak (*ρ* ranging from −0.103 to 0.321). The strongest nominal association was observed between HADS‐D and vallecular residue severity (*ρ* = 0.321; unadjusted *p* = 0.023), but this association did not remain significant after FDR correction (*p*_FDR_ = 0.368).

### 3.6 Additional Exploratory Analyses

SLT receipt was not significantly associated with subjective swallowing burden or swallowing‐related quality of life in the cohort (SSQ: *ρ* = 0.227, *p* = 0.112; MDADI: *ρ* = −0.271, *p* = 0.057).

Time since completion of oncological treatment was not significantly associated with subjective swallowing function as assessed by the SSQ total score (*ρ* = −0.219, *p* = 0.127; *n* = 50).

### 3.7 Multiple Regression Models

In rank‐transformed multiple linear regression analyses, HADS total score and PAS max were identified as independently associated factors of subjective dysphagia (SSQ total score). The overall model was statistically significant (*R* ^2^ = 0.455, adjusted *R* ^2^ = 0.420, *F*(3, 46) = 12.818, *p* < 0.001). Higher psychological distress (HADS total score: *β* = 0.372, *p* = 0.012) and greater penetration–aspiration severity (PAS max: *β* = 0.318, *p* = 0.006) were independently associated with higher SSQ scores. Although XI showed a positive coefficient, its independent association did not reach statistical significance (*β* = 0.268, *p* = 0.065).

For swallowing‐related quality of life, rank‐transformed multiple linear regression analysis identified significant independent associations of HADS total score, XI, and PAS max with MDADI total score. The overall model was statistically significant (*R* ^2^ = 0.593, adjusted *R* ^2^ = 0.566, *F*(3, 46) = 22.299, *p* < 0.001). Higher psychological distress (HADS total score: *β* = −0.339, *p* = 0.008), greater xerostomia (XI: *β* = −0.433, *p* < 0.001), and increased airway impairment (PAS max: *β* = −0.301, *p* = 0.003) were independently associated with reduced swallowing‐related quality of life. The regression coefficients and 95% confidence intervals for the SSQ and MDADI models are summarized in Figure 3.

**FIGURE 3:**
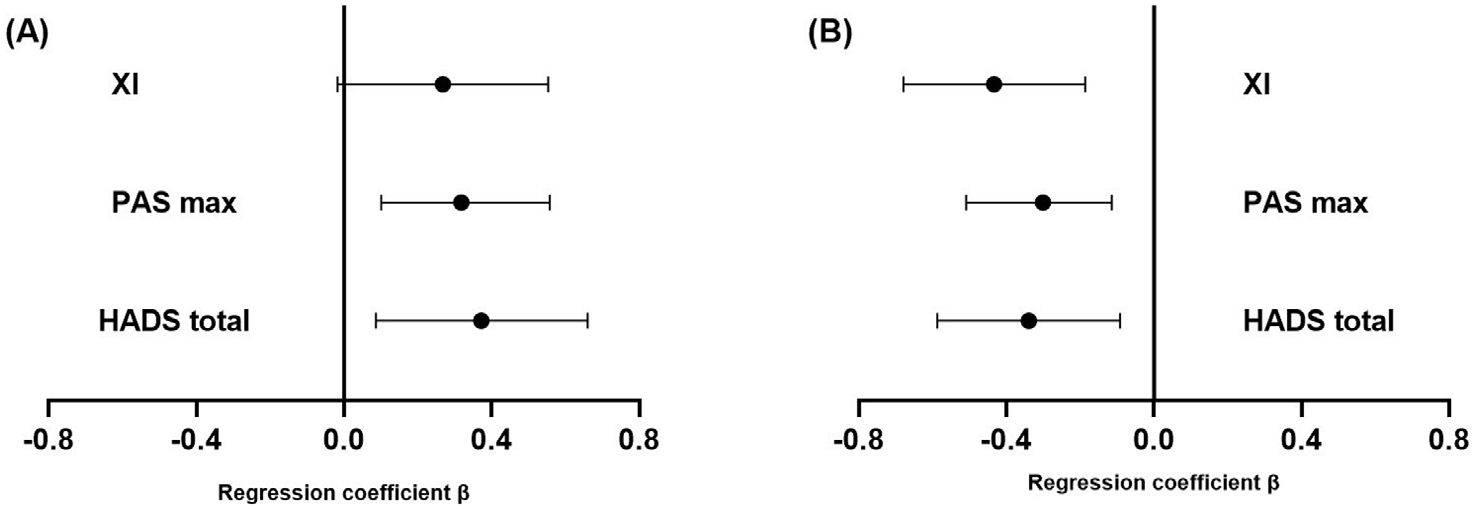
Forest plots of rank‐transformed multivariable regression models for patient‐reported swallowing outcomes. Forest plots showing standardized regression coefficients (*β*) and 95% confidence intervals from rank‐transformed multivariable linear regression models for SSQ total score (A) and MDADI total score (B). Both models included HADS total score, PAS max, and Xerostomia Inventory (XI) score. Higher SSQ scores indicate greater subjective dysphagia, whereas higher MDADI scores indicate better swallowing‐related quality of life. Dots indicate regression coefficients, and horizontal lines indicate 95% confidence intervals. The vertical reference line represents no association.

## 4 Discussion

The aim of this multicenter exploratory study was to investigate the relationship between objective swallowing function and patient‐reported dysphagia, as well as swallowing‐related quality of life, in patients with oropharyngeal following surgery‐based multimodal treatment with microvascular flap reconstruction. In addition, the role of psychological distress and xerostomia was examined in this context.

In the FDR‐adjusted Spearman correlation analyses, objective FEES swallowing parameters showed no significant associations with subjectively perceived dysphagia or swallowing‐related quality of life. In contrast, psychological distress and xerostomia were significantly associated with patient‐reported outcomes. The lack of significant associations between objective swallowing parameters and the subjective perception of dysphagia (SSQ and MDADI) is consistent with previous studies in patients with HNSCC. These studies reported only weak or inconsistent correlations between instrumentally measured objective swallowing findings and the symptoms reported by patients [4, 9, 10, 11, 13]. Several of these studies included broader clinical populations spanning different primary tumor sites, tumor stages and/or treatment modalities, whereas the present study focused on a more narrowly defined cohort of surgically treated patients with OPSCC who underwent radial forearm free‐flap reconstruction and adjuvant therapy. In addition, psychological distress and xerostomia were also assessed, unlike in the other studies.

In the study by Delaney et al., no consistent agreement between objective swallowing parameters and PROMs could be demonstrated [10]. Similarly, the study by Costa et al. showed limited agreement between subjective dysphagia symptoms and objectively assessed swallowing parameters in HNSCC patients after completion of therapy [9]. A possible explanation for the lack of association in the Spearman rank correlation may be that FEES primarily assesses swallowing safety and swallowing efficiency under standardized examination conditions. Subjective aspects such as swallowing effort, the sensation of residue, fear of aspiration, or adaptive eating behavior are not captured by this method. Particularly in surgically reconstructed OPSCC patients, anatomical changes, sensory disturbances, and functional compensatory mechanisms may be present that cannot be sufficiently captured in an objective FEES examination.

Hedström et al. reported only limited associations between objective PAS scores and subjective dysphagia [11]. This is consistent with our bivariate analyses, in which the weak nominal correlations of PAS max with SSQ and MDADI did not remain statistically significant after Benjamini–Hochberg correction, and no other FEES parameter was significantly associated with either outcome. In the exploratory rank‐transformed multivariable models, however, PAS max was associated with both SSQ and MDADI after adjustment for HADS total and XI. This suggests that objective signs of impaired swallowing safety, particularly penetration and aspiration, may indeed be relevant for patient‐reported outcomes. The results should therefore not be interpreted as contradictory, but rather as an indication of the multidimensional nature of patient‐reported dysphagia.

There was a significant association between psychological distress and subjective dysphagia as well as swallowing‐related quality of life in our study cohort. Both anxiety symptoms and depressive symptoms measured by HADS were associated with a higher subjective dysphagia burden (SSQ) and lower swallowing‐related quality of life (MDADI). It is known that psychological factors have a strong influence on patient‐reported outcomes in HNSCC [24]. One possible explanation is that psychological factors such as anxiety and depression influence or amplify the subjective perception of swallowing difficulties. Anxiety may lead to increased focus on the act of swallowing and its potential risks, such as aspiration, whereas depressive symptoms may be associated with an overall more negative assessment of one's own functional capacity. Future studies should further investigate this in a longitudinal design.

Furthermore, our study population showed a significant association between xerostomia and both increased subjective dysphagia burden and reduced swallowing‐related quality of life in the bivariate analyses. Xerostomia was associated with higher SSQ and lower MDADI scores in the bivariate analyses. In the mutually adjusted models, XI remained associated with MDADI, whereas its association with SSQ did not reach the conventional significance threshold. Xerostomia is one of the clinically most relevant long‐term side effects of adjuvant radiotherapy. Saliva plays an important role in oral bolus formation and processing [25]. These oral‐phase functions cannot be directly assessed by FEES. Furthermore, it is known that xerostomia significantly affects quality of life [26]. These results could suggest that xerostomia‐related symptoms have an independent influence on subjective swallowing parameters, which cannot be captured by objective FEES examination.

SLT was not significantly associated with subjective dysphagia burden or swallowing‐related quality of life in the investigated cohort. However, there was a non‐significant tendency toward higher SSQ scores and lower MDADI scores in patients who had received SLT. This should not be interpreted as an indication of an unfavorable effect of SLT. It can be assumed that patients with more pronounced swallowing complaints or greater clinical need for support were more frequently referred for SLT.

Limitations must be considered when interpreting the results. The study had a cross‐sectional study design. Longitudinal studies are required to investigate temporal dynamics. FEES does not directly visualize the oral or esophageal phases of swallowing. Impairments in these phases may contribute to patient‐perceived dysphagia but were not captured by the FEES‐derived parameters analyzed in this study. This limitation may partly explain the incomplete correspondence between FEES findings and patient‐reported swallowing outcomes. Furthermore, it should be considered that the assessment of pharyngeal residues using YPRSRS and BRACS does not account for adaptive eating behavior or avoidance strategies. Generalizability to other HNSCC subsites, primary radio‐/radiochemotherapy without surgery, or other reconstruction methods may be limited due to the highly selected nature of the study cohort. Another limitation is the variability in the interval between completion of oncological treatment and study data collection, which ranged from 3 to 112 months. Although no significant cross‐sectional association between time since treatment and subjective swallowing function as assessed by the SSQ was observed, longitudinal studies with repeated assessments are required to characterize changes in swallowing perception over time. In addition, future studies with larger cohorts should investigate whether specific patient‐reported symptoms or PROM domains are preferentially associated with greater penetration–aspiration severity, which may help identify the symptoms patients perceive when objective airway invasion is present. Finally, no formal a priori power‐based sample‐size calculation was performed. Although the prespecified recruitment target of 50 patients was achieved, the sample size may have limited the ability to detect weak associations, particularly after correction for multiple comparisons. Therefore, the findings, especially those from the multivariable analyses, should be interpreted as exploratory.

A strength of this study is the integrated analysis of instrumental swallowing findings, dysphagia‐specific PROMs, psychological distress, and xerostomia in a multicenter, clinically well‐defined reconstructive OPSCC cohort. Notably, comparative HNSCC studies did not include validated measures of psychological distress or xerostomia. Kirsh et al. [13] explicitly identified psychological health as an unassessed variable warranting future multivariable analysis, and Costa et al. [9] similarly called for the investigation of emotional and psychological factors alongside swallowing measures. Delaney et al. [10] incorporated a mental health domain only as part of a composite patient‐reported outcome score, precluding its use as an independent covariate. Hedström et al. [11] and Liou et al. [4] did not assess psychological or xerostomia‐related symptoms at all. The present study addresses this gap by incorporating HADS and XI.

Our data suggest that the FEES‐derived parameters assessed in this study do not fully reflect patient‐reported dysphagia in this specific cohort. The observed associations of xerostomia and psychological distress with subjective dysphagia and swallowing‐related quality of life support a multidimensional understanding of post‐treatment swallowing complaints in OPSCC patients after surgery‐based multimodal therapy and microvascular reconstruction.

## 5 Conclusion

To our knowledge, this study is the first to indicate, in this specific patient cohort, that objectively measured FEES parameters only partially capture patient‐reported dysphagia and swallowing‐related quality of life. While nominal associations of PAS max with SSQ and MDADI were observed in the bivariate correlation analyses, neither remained significant after FDR correction. However, PAS max was associated with both outcomes in exploratory multivariable models. In addition, psychological distress and xerostomia showed significant associations with the patient‐reported outcomes. These findings support a multidimensional understanding of post‐treatment dysphagia and the routine inclusion of psychosocial and symptom‐oriented parameters into follow‐up care in this complex patient cohort.

## Funding

The authors have nothing to report.

## Ethics Statement

The study was approved by the ethics committee of University Witten/Herdecke (ethics application number: S‐322/2025) and was conducted in accordance with the latest version of the Declaration of Helsinki.

## Conflicts of Interest

The authors declare no conflicts of interest.

## Data Availability

The datasets generated and analyzed during the current study are available from the corresponding author upon reasonable request, subject to applicable ethical and data protection requirements.
